# A Study in Insanity

**Published:** 1899-09-23

**Authors:** 


### A Study in Insanity.

WAS DR. HIERONIMUS' PATIENT INSANE?

"Professor Hieronimus" is, as many of our readers may know, the title of a remarkable novel which has, in Denmark, and indeed in Scandinavia generally, created some sensation. This novel—the work of a woman, Fru Amalie Skram, who has been declared by Björnsen, the novelist-politician, to be, with the exception of Zola, the only genuine "naturalist" in Europe—has recently been translated into English, and has already attracted no little attention. "Professor Hieronimus" has no sentimental interest; it has no plot; it conducts its characters to no happy destiny; it is anything but what young ladies call a "jolly story." It aims at being a kind of literary cinematograph; at reproducing a series of episodes in the life of Fru Kant, first in her husband's home, afterwards in the private sanatorium of Professor Hieronimus, the great specialist in mental disease. The photographs are vivid and impressive, but, to continue the metaphor, the lens with which they are taken is aberrant and faulty. The optic planes are untrue. The book has, indeed, no objective value such as Björnsen ascribes to it; its interest lies in the revelation of the workings of an essentially irrational mind.

Till quite recently, at all events, there was in Denmark but one private institution for the treatment of mental disease; the director, a man of strongly-marked character, has much in common with Hieronimus. It is well known, nay forced upon us, that Fru Skram was for some time a voluntary boarder in this institution under circumstances which called for rest and quiet. It is not surprising then that public opinion in Denmark has identified "Hieronimus" with the director of this sanatorium, and has considered the work as an exposure of his methods. Others—adopting the argument from the particular to the general so beloved of neuropaths and faddists—with Björnsen see in "Hieronimus" an argument against "the too arbitrary powers of asylums." But whether the motive of the authoress be artistic or polemical it can hardly be maintained that a lady requiring treatment in an asylum is in a condition accurately and dispassionately to reproduce on paper her experiences. It is obvious enough that the experiences of Fru Kant are those which Fru Skram believes to have been her own. They therefore deserve attention and consideration. Fru Skram, both directly and indirectly, protests the sanity of her heroine; in the alternative, as a lawyer would say, she pleads the total incompetency of the medical advisers, and protests against what she considers the despotic powers conceded to them. We are not concerned to deny—it would be idle so to do—that the lunacy laws of Denmark are less stringent than those of England. In fact, it is not improbable that the extreme stringency of lunacy law in England is, indeed, responsible in part for the admitted failure of our profession to cope with the advancing tide of insanity. And it may well be that in countries such as Denmark, where scientific opinion is notoriously respected, that physicians are able to make treatment of the insane a real thing, and not the matter of red-tape and official envelopes to which English "philanthropists" seek to reduce it. But it must be remembered that Fru Kant in the story, as Fru Skram in reality, sought treatment voluntarily at Hieronimus' hands.

Fortunately for literature, the activity of Fru Skram is a happy result of "Hieronimus'" treatment, harsh as it may have seemed to the lady at the time. But in the tale Fru Kant is removed to a public asylum, apparently on the mere assurance of her madness by word of mouth, and with no hint of judicial sanction. This we are compelled to call a monstrous misrepresentation. In Denmark, as in England, certification is necessary; State control is active; and permanent incarceration on the word of a doctor is quite impossible. But was Fru Kant sane, as the authoress protests? We think not. We believe that, unconsciously it may be, Fru Skram has enriched not only literature, but even medical science, by an intensely powerful and intimately subjective analysis of the mind of a woman, talented and neurotic, passing gradually from the "borderland" to the region of defined insanity. Let us consider the case of Fru Kant as we would that of a case in real life. We are made acquainted with a young married lady of marked ability who combines with her housewifely duties the pursuits of an artist to whom the morbid, mystic, and symbolic has a confessed attraction. This lady has suffered much anxiety during the care of her ailing and only child. At some former time she has suffered from insomnia and alienation. At this time she, we are told, did not recognise her nearest relatives. She had visual hallucinations; saw "a little fat grey man who was continually jumping up and down on the table, getting the lamp between his legs." Clearly Fru Kant has had at some time an attack of subacute mania. Yet Fru Skram, with the usual perversity of "la nèvreuse," explains this illness away on the rather lame grounds that it was in the first place due to "mental shock," and then that it was cured "by a dear, kind, gentle old doctor." At the time at which we make Fru Kant's acquaintance she has suffered during many months from a recurrence of the insomnia; she has a nervous cough, for which she constantly takes opiates; she begins to feel herself incompetent and her work a failure; she is tormented with remorse at the fall of a servant whom she has dismissed for theft, and holds herself responsible for the girl's ruin. She rises and paints in the small hours; sings and sobs about the house; refuses all medicine but her opiates; talks of suicide, and complains of the usual pains at the nape of the neck and at the vertex. Then she sees in her bedroom processions of "beautiful brown horses with stiff heads and black eyes," and the next day rows of coffins, corpses, and bedsteads. Little wonder, surely, that the sorely-tried husband in his love seeks advice and urges treatment by the great specialist Hieronimus. Even Else Kant herself recognises at length her mental perversity, and goes, not unwillingly, as a private patient to Hieronimus' sanatorium. So far the book is inimitable, and even the authoress implies occasionally a doubt of her heroine's perfect soundness. But with Fru Kant's admission to the sanatorium the tone changes. Else is no longer the wayward, half-insane woman, but an injured and persecuted being. The book, in fact, becomes little more than an expanded and brilliant version of such letters as the Commissioners in Lunacy daily receive in shoals. Animus is evident in every phrase. A door is locked; Else shivers and feels she is in prison. Of course, all the doors shut with a "clang"; cold, hard nurses rattle their keys, and the ordinary single room is never aught but a cell. Else—a suicidal patient be it remembered—draws from her pocket a vial and raises it to her lips. Of course, the harsh and brutal nurses seize the bottle. It contains an opiate—for the nervous cough. Else Kant cries, sobs, and storms. Much trouble ensues when Else desires to sleep in her stockings and garters. The nurses very properly object, and there are more hysterics. Yet this is Fru Skram's indictment of asylum routine: that suicidal patients are not allowed to sleep alone, with strong garters and with opiates at their command! However, the vials of Fru Skram's wrath are reserved for the doctors—in chief for Hieronimus; in a lesser measure for a luckless clinical assistant who, in his brutal way, "hammers" on Else's chest and coarsely bids her cheer up. He even orders a sleeping draught—poor, inconsiderate man—and, as we expected, this draught proves to be a "nauseous mixture," which Else, in proof, we suppose, of her sanity, refuses to take.

The character of Hieronimus is drawn with bitter skill and mordant hate. It so happens that we are intimately acquainted with an English lady who some few years ago was an inmate of the sanatorium conducted by the prototype of Hieronimus, and this lady is, or was, in the habit of bringing charges not dissimilar to those of Fru Skram. But we are satisfied that in each case the charges have no more basis than those commonly brought by a certain type of unsound humanity—or, perhaps, femininity—against asylum superintendents. Heironimus and his prototype very properly refused their respective patients access to their friends, and denied them the excitement of correspondence. The treatment was, in fact, a kind of modified Weir-Mitchell treatment. In the case of our friend the result was in great measure satisfactory, and if, as we are led to believe is the case, Fru Skram has in her book detailed her own experiences, we regret that she should have allowed herself to attack the man whose methods so happily restored her to society and literature. But in the tale Else goes from bad to worse. She rebels at every step, refuses all aid, calls her devoted attendants "executioners," and finally writes the usual insane letter to Hieronimus, signing herself "your sincere enemy." We have a boxful of such letters from lunatics before us as we write these lines. Small wonder that Hieronimus is forced to declare to Kunt Kant his wife's undoubted insanity, and to recommend detention in a public asylum. As Hieronimus succinctly declares, the marked feature of her insanity is total inability to control the emotional side of her nature. With Else's very proper removal to a public asylum the story abruptly ends.

Again we say that we will not deny the comparative laxity of procedures under the Danish lunacy laws. But we protest emphatically against the endorsement given by publicists such as Björnsen to a writer whose animus allows her to hint that nothing more than the *ipse dixit* of a single physician is required that a woman may be condemned to indefinite or perpetual incarceration in a public asylum. That "Hieronimus" has faults of character and temper is likely enough, but Fru Skram has totally failed to bring forward any damaging evidence against asylums in general, or that of Hieronimus in particular. It may be that Fru Kant could have been more suitably treated in private care. But the care of lunatics as single patients is a method for which lunacy reformers have always reserved their most scathing denunciations. We may, perhaps, reassure the timid by telling them, what should be common knowledge, that in England no person can be detained without elaborate medical examination and the intervention of a judicial authority, and that every inmate of an asylum is from the first the subject of a perfectly appalling mass of reports and official correspondence, and is jealously safeguarded in every way by an official Board and a whole series of visitors, County Councillors, Guardians of the Poor, Magistrates, and what not.

We have no intention of dwelling in detail on some of the really brilliant pieces of descriptive writing in Fru Skram's book. It is truly a reflection of asylum life, but it is like a reflection in a convex mirror. We cannot, however, withhold our thanks to Fru Skram for her really wonderful—though, as we believe, quite unconscious—analysis of an unsound mind, and for her subjectively faithful portrayal of a type of insanity that above all others brings pain and distress to the relatives of the afflicted one, and anxiety and stress to the medical attendants.

